# Knowledge of Clinical Trials Among US Cancer Survivors: Cross-Sectional Study of HINTS-SEER Data

**DOI:** 10.2196/76187

**Published:** 2025-11-19

**Authors:** Aisha Tene Langford, Katrina Renee Ellis, Nancy Buderer, Navreet Singh

**Affiliations:** 1 Department of Family Medicine and Public Health Sciences, Wayne State University, 6135 Woodward Ave, Detroit, MI, 48111, United States, 1 3135775644; 2 University of Michigan, Ann Arbor, MI, United States; 3 Nancy Buderer Consulting, LLC, Oak Harbor, OH, United States

**Keywords:** cancer survivors, clinical trials as topic, cross-sectional studies, health literacy, information-seeking behavior, social media, sociodemographic factors

## Abstract

**Background:**

Clinical trials are important for all stages of the cancer control continuum, including cancer survivorship.

**Objective:**

The purpose of this study was to evaluate correlates of general clinical trial knowledge among US adult cancer survivors.

**Methods:**

We conducted a cross-sectional analysis of the National Cancer Institute’s 2021 Health Information National Trends Survey. Cancer survivors were recruited from 3 Surveillance, Epidemiology, and End Results registries: Iowa Cancer Registry, Greater Bay Area Cancer Registry, and New Mexico Tumor Registry. Data collection occurred from January 11 to August 20, 2021. Eligible participants had a cancer diagnosis prior to 2018. The primary outcome was self-reported knowledge of clinical trials, assessed by the question: “*How would you describe your level of knowledge about clinical trials?*” Responses were dichotomized as knowing “a lot” or “a little bit” versus “don’t know anything.” Independent variables included sociodemographic characteristics, patient-centered communication, health information seeking (including watching health-related videos on YouTube), and confidence in obtaining cancer-related information. We used survey-weighted logistic regression to examine univariable and multivariable associations with clinical trial knowledge. A total of 2 a priori hypotheses were specified: (1) cancer survivors with a higher perceived quality of patient-centered communication would have greater knowledge of clinical trials than those with a lower perceived quality of patient-centered communication and (2) cancer survivors who were “completely confident” in their ability to obtain cancer-related information would have greater knowledge of clinical trials than those less confident. Odds ratios (ORs), 95% CIs, and *P* values were estimated using SAS (version 9.4; SAS Institute Inc, Cary, NC, USA).

**Results:**

Among cancer survivors (N=1207) included in the analysis, 269 (22.3%) reported that they did not know anything about clinical trials, while 938 (77.7%) reported knowing “a lot” or “a little.” Neither of the 2 a priori hypotheses was supported. In the multivariable weighted logistic regression model, greater knowledge of clinical trials was significantly associated with non-Hispanic White race compared with all other races (OR 2.55, 95% CI 1.59, 4.08; *P*<.001), having a college degree compared with less than a college degree (OR 3.50, 95% CI 2.25, 5.46; *P*<.001), seeking cancer information from any source (OR 3.04, 95% CI 2.10‐4.40; *P*<.001) compared with not, and ever watched health-related videos on YouTube (OR 2.71, 95% CI 1.49‐4.94; *P*=.002) compared with never watched. In contrast, female sex assigned at birth was associated with lower odds of clinical trial knowledge compared with male sex assigned at birth (OR 0.57, 95% CI 0.41‐0.80; *P*<.001).

**Conclusions:**

Sociodemographic characteristics and health-seeking behaviors including watching health-related videos on YouTube were associated with clinical trial knowledge among cancer survivors. These findings highlight opportunities to leverage YouTube as a platform to promote clinical trial awareness and to strengthen survivors’ cancer-specific information-seeking skills to improve access to clinical trial information.

## Introduction

### Background and Rationale

Clinical trials test new ways to identify, prevent, and treat cancer via diagnostic, behavioral, medical, surgical, and other types of interventions (eg, gene therapy) [1,2]. Over time, increased attention has been focused on improving clinical trial participation [3] and increasing age, sex, and racial or ethnic representation in clinical trials [4-6].

### Factors Influencing Clinical Trial Participation

While many factors contribute to differences in general clinical trial participation, lack of knowledge and awareness about clinical trials is a significant barrier to recruitment [1,7-9]. That is, people cannot participate in a clinical trial if they do not know about the option of joining a clinical trial in the first place or do not have some level of health literacy to support informed decision-making about clinical trial participation. *Healthy People 2030* defines personal health literacy as “the degree to which individuals have the ability to find, understand, and use information and services to inform health-related decisions and actions for themselves and others” [10]. Prior research on cancer information seeking has demonstrated that patient-provider communication scores, self-efficacy in managing one’s own health, difficulty understanding information, concern about the quality of information, and frustration during the search for information are associated with self-efficacy for obtaining cancer information [11,12].

Naturally, health literacy and cancer information-seeking skills can directly influence knowledge about clinical trials, especially because many clinical trial opportunities are posted online. Strategies to enhance general clinical trial knowledge about cancer and other medical conditions include media campaigns [13,14], clinical trial registries such as ResearchMatch [15], websites and online databases such as ClinicalTrials.gov [16], decision aids [17,18], tailored and targeted messages about research participation [19,20], direct-to-consumer and direct-to-patient invitations [21-23], patient education materials [24,25], and group-based information sessions [26,27].

### Clinical Trial Knowledge and Awareness Among Cancer Survivors

Regarding cancer survivors’ knowledge of clinical trials and willingness to participate in research, differences by race or ethnicity have been observed. Using cross-sectional data from 147 Black and White cancer survivors who took the “Minority Patient Participation in Cancer Clinical Trials” survey, Kumar et al [28] found that Black cancer survivors were less likely than White cancer survivors to be aware of cancer clinical trials, express willingness to participate in cancer clinical trials, and participate in cancer clinical trials. Using data from the 2020 Health Information National Trends Survey, Commaroto et al [1] described cancer survivors’ (n=553) knowledge of clinical trials and prior invitations to participate in clinical trials. They found that cancer survivors with a high school diploma were less likely to have any knowledge of clinical trials than those with a baccalaureate degree or higher. However, no statistically significant differences were found in clinical trial knowledge or prior invitation to participate by sex, age, or race or ethnicity. Lastly, Cho et al [29] evaluated data from 958 cancer survivors from the 2021 Health Information National Trends Survey-Surveillance, Epidemiology, and End Results (SEER) Program and found that having 1 or more comorbidities was significantly associated with lower rates of clinical trial discussions but not clinical trial participation. While these studies provide useful information, it is also important to explore the correlates of clinical trial knowledge of cancer survivors that include but extend beyond sociodemographic factors.

### Present Study

The purpose of this study was to evaluate the associations between knowledge of clinical trials among US cancer survivors and perceived quality of patient-centered communication, cancer information seeking, emotional support, and watching health-related videos on YouTube. A priori, we proposed 2 main hypotheses:

Hypothesis 1: Cancer survivors with a higher perceived quality of patient-centered communication would have greater knowledge of clinical trials than those with a lower perceived quality of patient-centered communication.

Hypothesis 2: Cancer survivors who were “completely confident” that they could get advice or information about cancer if they needed it would have a greater knowledge of clinical trials than those who are less than completely confident.

Greater insights into ways to enhance clinical trial awareness among cancer survivors can support ongoing efforts to improve equitable participation and inclusion in cancer clinical trials.

## Methods

### Overview of the Health Information National Trends Survey

The Health Information National Trends Survey (HINTS) regularly collects nationally representative data on the American public’s knowledge of, attitudes toward, and use of cancer and health-related information [30]. The HINTS data collection program was created to monitor changes in the rapidly evolving field of health communication [31]. In 2021, the National Cancer Institute undertook a pilot project to oversample cancer survivors for the HINTS using 3 cancer registries from the SEER Program as a sampling frame for cancer survivors: Iowa Cancer Registry, Greater Bay Area Cancer Registry, and New Mexico Tumor Registry [32]. HINTS-SEER data can be accessed by submitting a data request through the HINTS website, where comprehensive methodology details are also available. All HINTS-SEER data are deidentified. HINTS-SEER data were collected from January 11, 2021, through August 20, 2021. Eligibility criteria were as follows: cancer diagnosis specified to invasive cancers, 18 years of age and older, last contact no earlier than January 1, 2016, and a diagnosis date prior to 2018 based on certified data. Survivors who were only diagnosed with nonmelanoma skin cancer were excluded. However, survivors diagnosed with nonmelanoma skin cancer, in addition to other cancers, were included.

The present study used HINTS SEER data to cross-sectionally explore the associations between knowledge of clinical trials and various sociodemographic factors. A total of 27 of the 1234 responses were removed from the data analysis because of a missing response on the dependent variable of interest (knowledge of clinical trials), leaving a final sample size for analysis of 1207.

### Ethical Considerations

This study used publicly available, de-identified HINTS-SEER data to conduct a secondary analysis. The data contain no personal identifiers. Because the dataset is publicly available and deidentified, this research does not involve human subjects as defined by federal regulations (45 CFR 46.102) and is exempt from institutional ethics review. Accordingly, Institutional Review Board (IRB) review at the first author’s institution was not required. It should be noted that a formal Micro-Data Dissemination Policy for the Health Information National Trends Survey: HINTS SEER data use agreement between the author’s home institution and HINTS was signed on October 23, 2023. The parent HINTS-SEER pilot study underwent expedited review by the Westat IRB and was approved as an amendment to HINTS 5 on May 22, 2020 (Project Number 6048.14, Amendment ID #3212). The participating SEER registries obtained independent IRB approvals for their participation in the study. The full details of the selection of registries, the sampling and consenting procedures, and compensation can be found in the methodology report [33].

### Measures

The following survey items were used to conduct the analysis for this study.

*Knowledge of Clinical Trials (primary outcome*). First, participants read the following prompt: “Clinical trials are research studies that involve people. They are designed to compare new kinds of health care with the standard healthcare people currently get. For example, a new drug or a new way for patients to track their diets.” Next, participants were asked, “How would you describe your level of knowledge about clinical trials?” The response options were (1) I don’t know anything about clinical trials, (2) I know a little bit about clinical trials, and (3) I know a lot about clinical trials. For the primary analysis, we compared “I don’t know anything about clinical trials” with “know a lot” and “know a little bit” combined. As a secondary analysis, we compared survivors who reported that they know “a lot” with those who reported that they know “a little bit” about clinical trials.

### Sociodemographic Factors

*Age* was evaluated continuously and by group (18‐64 y, 65‐74 y, and 75+ y). *Race or Ethnicity* was evaluated as non-Hispanic White versus all other racial or ethnic categories combined: Black or African American, Hispanic, Asian, and Other. *Sex Assigned at Birth* options were male and female. *Education* was evaluated as college graduate versus not college graduate.

*Feelings about Household Income* were assessed with the question, “Which one of these comes closest to your own feelings about your household’s income?” The response options were (1) living comfortably on present income, (2) getting by on present income, (3) finding it difficult on present income, and (4) finding it very difficult on present income. We consolidated the difficulty-related answers such that 3 groups were evaluated in the analysis: “living comfortably” versus “getting by” versus “finding it difficult” or “finding it very difficult on present income.”

### Health Information Seeking, Trust, and Understanding

*Cancer Information Seeking*. Participants were asked if they had ever looked for information about cancer from any source. Response options were yes and no.

*Confidence to Get Cancer Health Information* was assessed with the item, “Overall, how confident are you that you could get advice or information about cancer if you needed it?” The response options were completely confident, very confident, somewhat confident, a little confident, and not confident at all. We compared “completely confident” responses with all other responses combined.

*Health Information Seeking on Social Media*. First, participants read the prompt, “Sometimes people use the Internet to connect with other people online through social networks like Facebook or Twitter.” This is often called “social media.” Next, they were asked, “In the last 12 months, have you used the Internet for any of the following reasons?” Response options were (1) to visit a social networking site, such as Facebook or LinkedIn; (2) to share health information on social networking sites, such as Facebook or Twitter; (3) to participate in an online forum or support group for people with similar health or medical issues; and (4) to watch a health-related video on YouTube. Given that YouTube is the most widely used social media platform [34] and a common way for people to get health information [35], we only evaluated responses to watching health-related videos on YouTube in this analysis.

*Trust Information From Doctor*. Participants were asked how much they would trust information about cancer from a doctor. The response options were a lot, some, a little, and not at all. We compared “a lot” responses to all the others combined.

*Understanding Information in Online Medical Records*. Participants were asked how easy or difficult it was to understand the health information in their online medical record. The response options were very easy, somewhat easy, somewhat difficult, and very difficult. We compared “very easy” with all other categories combined.

### Communication with Health Professionals

*Patient-Centered Communication Scale*. The Patient-Centered Communication Scale variable was created using 7 items related to communication. Specifically, participants were asked how often doctors, nurses, or other health professionals they saw during the past 12 months were as follows: (1) give you the chance to ask all the health-related questions you had, (2) give the attention you needed to your feelings and emotions, (3) involve you in decisions about your health care as much as you wanted, (4) make sure you understood the things you needed to do to take care of your health, (5) explain things in a way you could understand, (6) spend enough time with you, and (7) help you deal with feelings of uncertainty about your health or health care. The response options included always=1, usually=2, sometimes=3, and never=4. The values of these 7 variables were reverse-scored, such that never=1 and always=4. The mean of these 7 variables (if at least half of the variables had valid values) was generated. Finally, the mean value was transformed to a 0‐100 scale.

### Time Passed Since Final Treatment and Emotional Support

#### Time Since Last Cancer Treatment

Participants were asked, “About how long ago did you receive your last cancer treatment?” Response options were: still receiving treatment, less than 1 year ago, 1 year ago to less than 5 years ago, 5 years ago to less than 10 years ago, and 10 or more years ago. We evaluated the following 4 categories: still in treatment, <5 years ago, 5‐10 years ago, and ≥10 years ago or more.

#### Emotional Support

This was assessed using the question, “Is there anyone you can count on to provide you with emotional support when you need it, such as talking over problems or helping you make difficult decisions?” The response options were yes or no.

### Data Analysis

Odds ratios (ORs), 95% CIs, and *P* values were calculated using SAS (version 9.4; SAS Institute Inc, Cary, NC, USA) proc surveylogistic with jackknife weighting, including the overall weight and 50 replicate weights, and the Newton-Raphson algorithm. The results are presented as odds ratios [95% CIs] for the odds of having knowledge about clinical trials and *P* values. Due to the small sample size (<25), some response categories were combined. Factors that had a corresponding *P*<.10 in univariable models were attempted in the multivariable model. In univariable analyses, all available data were used for each factor. Surveys that were missing a response were simply excluded from consideration for that factor’s univariable analysis. Using backward elimination, factors were removed one by one until all the remaining factors in the model were significant (*P*<.05). It should be noted that a higher percentage of the California registry respondents had knowledge of clinical trials than respondents from the New Mexico registry. Therefore, we adjusted for registry in the final models. When interpreting ORs, CIs that do not include the value 1 are considered significant. Data were analyzed using SAS (version 9.4) and SAS/STAT (version 14.1; SAS Institute Inc). Given that we first compared (primary analysis) “I don’t know anything about clinical trials” with “know a lot or know a little bit” combined and secondarily compared “know a lot” versus “know a little bit,” the sample sizes varied across analyses.

## Results

**Figure 1.**
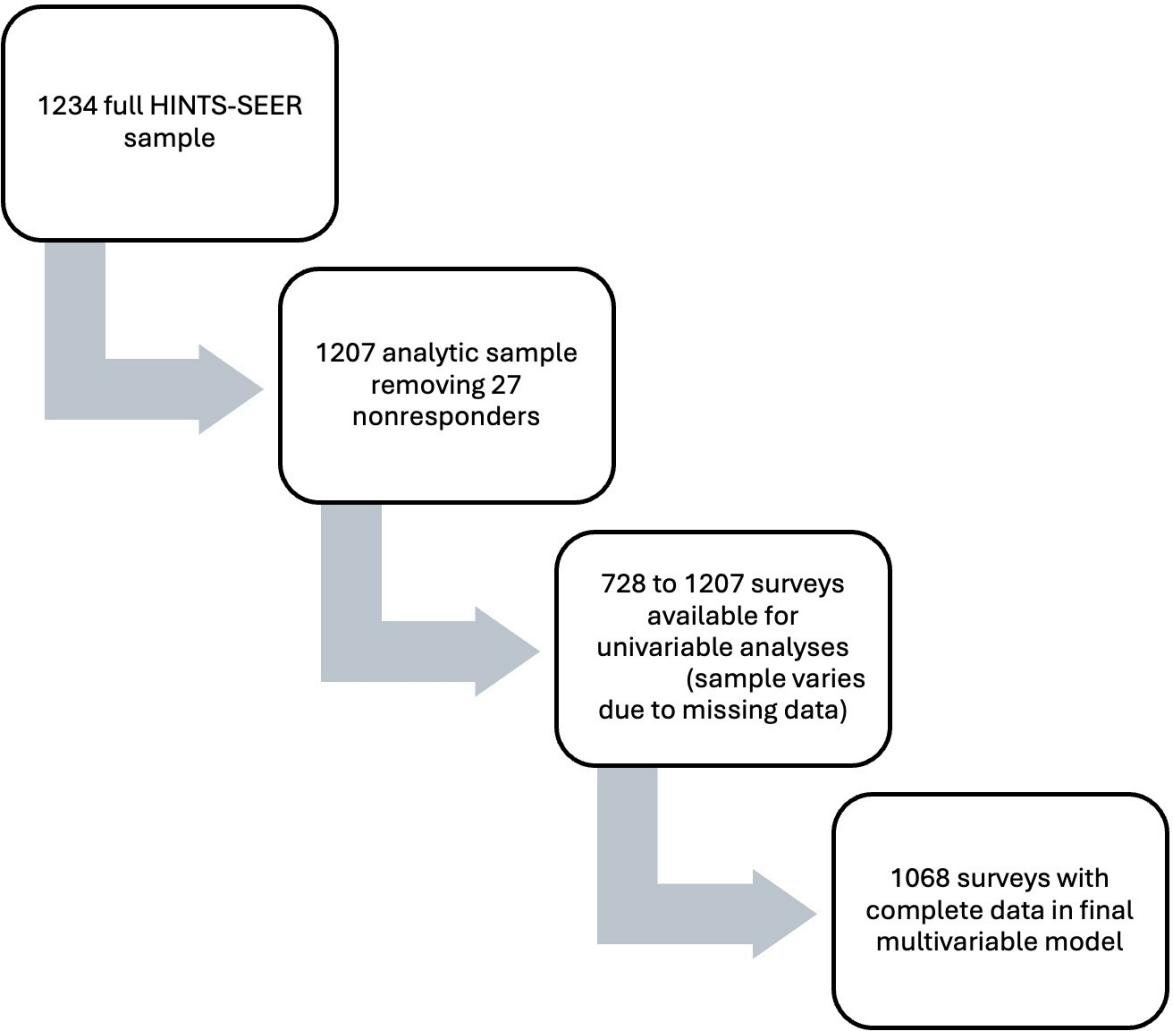
Creation of data set. HINTS-SEER: Health Information National Trends Survey-Surveillance, Epidemiology, and End Results.

Figure 1 shows the selection of the final sample size for the primary analysis. Table 1 provides counts and percentages (unweighted) for variables of interest. Overall, 486 (41.3%) of the sample were 75 years or older, 894 (80.0%) were non-Hispanic White, and 677 (57.3%) were college graduates.

**Table 1.** Demographic characteristics of the analytic sample (unweighted).

Characteristic		Clinical trials knowledge	
	All (n=1207)	Don’t know anything, n=269; n (%)	Know a lot or a little bit, n=938; n (%)
Age (mean years), n=1176	71.3 (11.5)	73.85 (11.36)	70.58 (11.50)
Age (y), n=1176			
18‐64	286 (24.3)	55 (19.23)	231 (80.77)
65‐74	404 (34.4)	69 (17.08)	335 (82.92)
75+	486 (41.3)	136 (27.99)	350 (72.02)
Race or ethnicity, n=1118			
Non-Hispanic White	894 (80.0)	156 (17.45)	738 (82.55)
All other races	224 (20.0)	81 (36.16)	143 (63.84)
Birth sex, n=1182			
Male	548 (46.4)	112 (20.44)	436 (79.56)
Female	634 (53.6)	149 (23.50)	485 (76.50)
Education, n=1182			
College graduate	677 (57.3)	77 (11.4)	600 (88.63)
Not college grad	505 (42.7)	182 (36.04)	323 (63.96)
Income feelings, n=1167			
Living comfortably on present income	752 (64.4)	119 (15.82)	633 (84.18)
Getting by	318 (27.3)	99 (31.13)	219 (68.87)
Finding it difficult or very difficult	97 (8.3)	36 (37.11)	61 (62.89)
Registry			
California (Bay Area)	468 (38.8)	86 (18.4)	382 (81.6)
Iowa	401 (33.2)	91 (22.7)	310 (77.31)
New Mexico	338 (28.0)	92 (27.2)	246 (72.8)

In weighted univariable analyses (Table 2), the following factors were associated with higher odds of having knowledge of clinical trials: White respondents (compared to all other races combined), college graduates (compared to not college graduate), living comfortably on present income (compared to finding it difficult or very difficult), having looked for cancer information from any source, having someone to count on for emotional support, and used the internet to watch a health-related video.

**Table 2.** Factors associated with clinical trials knowledge using the Health Information National Trends Survey-Surveillance, Epidemiology, and End Results (HINTS SEERS) dataset (weighted univariable analyses)^a^.

Factor	Clinical trials knowledge			
	Don’t know anything (n=269), % (SE)	Know a lot or a little bit (n=938), % (SE)	Unadjusted odds ratio for know a lot or a little [CI]	*P* value
Age (mean years), n=1176	74.07 (0.87)	69.49 (0.33)	0.97 [0.95, 0.99]	<.001
Age (y), n=1176				Overall *P*=.003
18‐64	17.41 (2.28)	82.59 (2.28)	Reference	—
65‐74	17.20 (2.55)	82.80 (2.55)	1.02 [0.64, 1.62]	.95
75+	28.70 (2.58)	71.31 (2.58)	0.53 [0.34, 0.80]	.003
Race or ethnicity, n=1118				
Non-Hispanic White	17.01 (1.15)	82.99 (1.15)	2.39 [1.68, 3.39]	<.001
All other races	32.87 (3.76)	67.13 (3.76)	Reference	—^b^
Birth sex, n=1182				
Male	19.40 (2.03)	80.60 (2.03)	Reference	—
Female	24.16 (1.74)	75.84 (1.74)	0.76 [0.55, 1.03]	.07
Education, n=1182				
College graduate	11.50 (1.36)	88.50 (1.36)	4.43 [3.08, 6.37]	<.001
Not college graduate	36.51 (2.63)	63.49 (2.63)	Reference	—
Income feelings, n=1167				Overall *P*<.001
Living comfortably on present income	16.40 (1.52)	83.60 (1.52)	2.67 [1.64, 4.36]	<.001
Getting by	30.34 (2.63)	69.66 (2.63)	1.20 [0.68, 2.12]	.52
Finding it difficult or very difficult	34.40 (5.55)	65.60 (5.55)	Reference	—
Looked for cancer information from any source, n=1188				
Yes	14.44 (1.05)	85.56 (1.05)	5.49 [3.85, 7.82]	<.001
No	48.07 (4.03)	51.93 (4.03)	Reference	—
Confident that you could get advice or information about cancer if you needed it, n=1179				
Completely confident	18.02 (2.41)	81.98 (2.41)	1.39 [0.96, 2.00]	.08
Very, somewhat, a little, or not at all confident	23.33 (1.61)	76.67 (1.61)	Reference	—
Trust information about cancer from a doctor, n=1176				
A lot	21.05 (1.42)	78.95 (1.42)	1.05 [0.61, 1.79]	.87
Some, a little, not at all	21.83 (4.18)	78.18 (4.18)	Reference	—
Patient centered communication scale (mean score), n=1105	82.14 (1.78)	83.09 (0.83)	1.00 [0.99, 1.01]	.63
Understand health information in your online medical record, n=728				
Very easy	11.07 (2.12)	88.93 (2.11)	1.43 [0.84, 2.44]	.19
Somewhat easy, somewhat difficult, very difficult	15.11 (2.12)	84.89 (2.12)	—	—
How long ago did you receive your last cancer treatment, n=1077				Overall *P*=.56
<5 years ago	20.56 (3.02)	79.44 (3.02)	Reference	—
5‐10	20.68 (2.75)	79.32 (2.75)	0.99 [0.61, 1.62]	.97
10 or more	18.79 (2.58)	81.21 (2.58)	1.12 [0.66, 1.88]	.67
Still in treatment	26.24 (4.87)	73.76 (4.87)	0.73 [0.39, 1.34]	.30
Anyone you can count on for emotional support, n=1202				
Yes	21.18 (1.50)	78.82 (1.50)	1.91 [1.17, 3.14]	.01
No	33.95 (5.05)	66.05 (5.05)	Reference	—
In last 12 months, used the internet to watch a health-related video, n=1166				
Yes	10.08 (2.33)	89.92 (2.33)	3.27 [1.80, 5.94]	<.001
No	26.84 (1.86)	73.16 (1.86)	Reference	—
Registry, n=1207				Overall *P*=.02
California (Bay Area)	19.15 (2.11)	80.85 (2.11)	1.68 [1.18, 2.39]	.005
Iowa	25.05 (2.34)	74.95 (2.34)	1.19 [0.85, 1.67]	.31
New Mexico	28.47 (2.42)	71.53 (2.42)	Reference	—

a Row percentages are weighted. SE is the weighted standard error. Odds ratio is weighted, univariable (ie, not adjusting for any other variable). *P* value is weighted logistic regression *P* value. CI is a weighted 95% confidence interval for the odds ratio. Reference is the reference category of the independent variable. Some missing data for each factor.

b indicates reference category.

 As shown in Table 3, the multivariable model adjusting for SEER-registry site revealed that White race, male sex, college graduate, looked for cancer information from any source, and having watched a health-related video on YouTube remained statistically significant and were associated with increased odds of having knowledge of clinical trials. Of the 1068 respondents in the table, 850 had knowledge of clinical trials and 218 did not. ORs and 95% CIs are weighted and are adjusted for all the other variables in the multivariable model. OR, CI, and *P* value are from weighted, multivariable logistic regression.

**Table 3.** Factors associated with clinical trials knowledge using the Health Information National Trends Survey Survey-Surveillance, Epidemiology, and End Results (HINTS SEERS) dataset—weighted multivariable model, adjusting for registry (n=1068).

Factor	Clinical trials knowledge	
	Adjusted odds ratio for know a lot or a little [CI]	*P* value
Race or ethnicity		
Non-Hispanic White	2.55 [1.59, 4.08]	<.001
All other races	Reference	—^a^
Birth sex		
Male	Reference	—
Female	0.57 [0.41, 0.80]	.001
Education		
College graduate	3.50 [2.25, 5.46]	<.001
Not college graduate	Reference	—
Looked for cancer information from any source		
Yes	3.04 [2.10, 4.40]	<.001
No	Reference	—
In last 12 months, used the internet to watch a health-related video		
Yes	2.71 [1.49, 4.94]	.002
No	Reference	—
Registry		Overall *P*=.34
California (Bay Area)	1.33 [0.88, 2.00]	.18
Iowa	1.09 [0.68, 1.75]	.72
New Mexico	Reference	—

a indicates reference category.

To further explore the relationships between awareness of clinical trials among cancer survivors, we also compared those reported knowing “a little bit (n=768)” versus those who reported knowing “a lot (n=170)” in a secondary analysis. Table 4 shows the univariable associations.

**Table 4.** Factors associated with clinical trials knowledge (know a lot vs know a little bit) - univariable models.

Factor	Clinical trials knowledge	
	Unadjusted odds ratio for know a lot compared to know a little bit [CI]	*P* value
Age (mean years), n=916	0.99 [0.97, 1.00]	.10
Age (y), n=916		Overall *P*=.08
18‐64	Reference	—^a^
65‐74	1.00 [0.56, 1.76]	.99
75+	0.62 [0.35, 1.11]	.10
Race or ethnicity, n=881		
Non-Hispanic White	1.32 [0.69, 2.53]	.39
All other races	Reference	—
Birth sex**,** n=921		
Male	Reference	—
Female	0.73 [0.50, 1.07]	.11
Education, n=923		
College graduate	3.39 [2.00, 5.76]	<.001
Not college graduate	Reference	—
Income feelings, n=913		Overall *P*=.67
Living comfortably on present income	0.74 [0.33, 1.62]	.44
Getting by	0.66 [0.26, 1.67]	.37
Finding it difficult or very difficult	Reference	—
Looked for cancer information from any source, n=927		
Yes	4.21 [1.94, 9.13]	<.001
No	Reference	—
Confident that you could get advice or information about cancer if you needed it**,** n=920		
Completely confident	2.08 [1.33, 3.25]	.002
Very, somewhat, a little, or not at all confident	Reference	—
Trust information about cancer from a doctor, n=924		
A lot	0.69 [0.43, 1.10]	.12
Some, a little, not at all	Reference	—
Patient- centered communication scale, n=875	1.01 [1.00, 1.02]	.03
Understand health information in your online medical record**,** n=631		
Very easy	2.11 [1.38, 3.23]	<.001
Somewhat easy, somewhat difficult, very difficult	Reference	—
How long ago did you receive your last cancer treatment, n=853		Overall *P*=.82
<5 years ago	Reference	—
5‐10	1.00 [0.60, 1.66]	>.99
10 or more	0.88 [0.50, 1.55]	.66
Still in treatment	0.74 [0.34, 1.64]	.45
Anyone you can count on for emotional support, n=935		
Yes	1.22 [0.59, 2.53]	.58
No	Reference	—
In last 12 months, used the internet to watch a health-related video, n=911		
Yes	1.65 [1.10, 2.47]	.02
No	Reference	—
Registry		Overall *P*=.68
California (Bay Area)	1.05 [0.67, 1.64]	.83
Iowa	0.86 [0.52, 1.41]	.54
New Mexico	Reference	—

a indicates reference category.

As shown in Table 5, the multivariable model revealed that higher odds of reporting knowing “a lot” about clinical trials among cancer survivors were associated with college graduates (OR 2.84, CI 1.40, 5.75), those who looked for cancer information from any source (OR 3.17, CI 1.13, 8.90), and those who found it very easy to understand the health information in their online medical record (OR 2.08, CI 1.35, 3.20).

**Table 5.** Factors associated with clinical trials knowledge (know a lot vs a little bit)—multivariable model, adjusting for registry (n=618).

Factor	Clinical trials knowledge	
	Adjusted odds ratio for know a lot versus know a little bit [CI]	*P* value
Education		
College graduate	2.84 [1.40, 5.75]	.005
Not college graduate	Reference	—^a^
Looked for cancer information from any source		
Yes	3.17 [1.13, 8.90]	.03
No	Reference	—
Understand health information in your online medical record		
Very easy	2.08 [1.35, 3.20]	.001
Somewhat easy, somewhat difficult, very difficult	Reference	—
Registry		Overall *P*=.19
California (Bay Area)	0.67 [0.41, 1.10]	.11
Iowa	0.62 [0.33, 1.15]	.13
New Mexico	Reference	—

a indicates reference category.

## Discussion

### Principal Findings

The objective of this study was to evaluate associations between knowledge of clinical trials among US adult cancer survivors and key correlates of interest. In the primary analysis comparing those who know “a lot” or a “little bit” combined versus those who did not know anything about clinical trials, we found that White race, a 4-year college education, and health information seeking including having watched a health-related video on YouTube were associated with increased odds of having knowledge of clinical trials, whereas female sex assigned at birth was associated with lower odds of having knowledge of clinical trials. Our first hypothesis that cancer survivors with a higher perceived quality of patient-centered communication would have greater knowledge of clinical trials than those with a lower perceived quality of patient-centered communication was not supported by the results. Moreover, our second hypothesis that cancer survivors who were “completely confident” that they could get advice or information about cancer if they needed it would have greater knowledge of clinical trials than those who are less than completely confident about finding cancer information if needed was also not supported by the results.

In the secondary analysis comparing those who know “a lot” versus “a little bit” about clinical trials, we found slightly different trends. In summary, those who were college graduates, those who looked for cancer information from any source, and those who understood health information in their online medical records had higher odds of knowing “a lot” about clinical trials.

### Health Information Seeking

A novel finding was that cancer survivors who watched health-related videos on YouTube had higher odds of clinical trial knowledge. Given YouTube’s broad reach, including among older adults [34], video-based education may be a scalable avenue for improving awareness of clinical trial opportunities. Several mechanisms may explain why watching health-related videos on YouTube is associated with greater clinical trial knowledge. First, YouTube provides a dynamic, visually engaging format that can simplify complex topics such as trial design, consent, and risks or benefits, potentially improving comprehension and retention. Second, YouTube’s algorithm can create an “information cascade,” where viewing 1 cancer-related video leads to recommendations of related content including clinical trial education [36]. Third, YouTube allows for peer-to-peer storytelling. Accordingly, testimonials from survivors or clinical trial participants may build trust and normalize clinical trial participation, which could be particularly impactful compared to static text-based resources that are often used to describe clinical trials. Finally, unlike passive browsing, intentionally watching health-related videos likely reflects higher digital health literacy and proactive health engagement; these traits may predict greater awareness of clinical trial opportunities.

Our results related to watching health-related videos on YouTube complement Nutbeam’s conceptualization of health literacy as not merely functional (basic reading and numeracy) but also interactive and critical [37,38]. Survivors who actively sought cancer information and who watched health-related videos on YouTube may be engaging in interactive or critical health literacy, leveraging digital platforms to deepen their understanding of research opportunities. Future interventions could therefore focus on improving survivors’ critical health literacy, particularly among those with lower educational attainment, to reduce disparities in clinical trial knowledge [39]. Clinicians and cancer centers could incorporate curated video content into survivorship care, and policies that encourage evidence-based digital health communication may help ensure content quality. Future research should identify optimal video characteristics (eg, length, storytelling approaches) that most effectively promote informed decision-making.

Other health information-seeking findings were that cancer survivors who ever looked for information about cancer from any source had higher odds of clinical trial knowledge, further underscoring the importance of health information-seeking skills. Health systems and patient navigators could play a key role in training survivors to locate and evaluate credible information, while policies supporting digital health literacy initiatives may help reduce disparities. Research should examine which strategies best sustain long-term information-seeking engagement.

### Sex Differences

Our finding that female sex assigned at birth was associated with lower clinical trial knowledge compared with male sex assigned at birth warrants further consideration. Although speculative, individuals assigned female at birth may engage more frequently with preventive and routine health care services yet experience different communication patterns during oncology visits, including shorter discussions about clinical research opportunities or lower referral rates to clinical trials. In addition, structural factors such as caregiving responsibilities and time constraints may limit opportunities to participate in research discussions or to seek supplemental information. Addressing these multifaceted factors through patient-centered communication strategies, tailored educational materials, and flexible clinical trial designs may help mitigate sex- and gender-related disparities in clinical trial knowledge and participation.

### Race or Ethnicity Differences

We observed that non-Hispanic White survivors were more likely to have clinical trial knowledge. Knowledge of clinical trials is needed before a cancer survivor can make an informed decision about participation. While many studies have shown that *willingness* and *intent to participate* in clinical trials do not vary across racial or ethnic groups if a clinical trial is explicitly offered to a person [20,40-42], it is imperative that health care systems and health professionals ensure equitable opportunities for cancer patients to be aware and knowledgeable about the clinical trials for which they may be eligible [43]. For example, oncologists can routinely present clinical trial opportunities to cancer survivors during medical encounters. eHealth interventions may also play a role in enhancing clinical trial knowledge [44], while direct-to-patient invitations via patient portals may enhance awareness of clinical trial opportunities without relying on health professionals to be gatekeepers of information [23]. Additionally, policies could support funding for inclusive recruitment and education campaigns.

### Education Differences

Our finding that survivors with a college degree were more likely to report higher clinical trial knowledge underscores the relationship between educational attainment and health literacy. General information seeking and health-specific information-seeking models posit that sociodemographic characteristics (eg, education) shape both the motivation to seek information and the skills required to evaluate and apply it [39,45-47]. Higher educational attainment is associated with stronger critical appraisal skills, greater self-efficacy for information seeking, and more frequent use of diverse information channels. These factors likely enhance survivors’ ability to locate and interpret complex information about clinical trials, aligning with evidence that health literacy mediates the effect of education on knowledge and decision-making [43]. Higher educational attainment being linked to greater clinical trial knowledge may also reflect better access to academic medical centers that offer more clinical trial opportunities than community hospitals or federally qualified health centers. Additionally, cancer survivors with more education may proactively seek clinical trial options on health system websites, the National Cancer Institute website, or ClinicalTrials.gov, which may enhance their general knowledge of clinical trials. Strengthening health literacy and providing simplified, accessible trial information across care settings may help mitigate this gap. Future work could evaluate interventions that promote trial awareness among survivors with less formal education.

### Comparison to Prior Work

Our study complements the research by Gerdes et al, who explored clinical trial knowledge, discussions, and participation among cancer survivors using HINTS-SEER data [48]. In summary, they found that approximately 78% of cancer survivors reported having little or a lot of clinical trial knowledge and that those who discussed clinical trials with a health care provider had 8.71 higher odds of clinical trial knowledge than those who did not discuss clinical trials with a health care provider. Additionally, they found that clinical trial knowledge was lower for Hispanic versus non-Hispanic White participants. This study builds on the work of Gerdes et al by providing new insights into clinical trial knowledge among cancer survivors through our comprehensive evaluation of different correlates, including general patient-centered communication, emotional support, cancer information-seeking behaviors, and the use of YouTube to watch health-related videos. Additionally, while other studies have used HINTS data to evaluate associations between watching health-related videos on social media and correlates, including perceived patient-centered communication [49] and human papillomavirus awareness [35], to our knowledge, no studies have exclusively evaluated the relationship between watching health-related videos on YouTube and clinical trial knowledge among cancer survivors.

### Strengths and Limitations

The strengths of this study include the large sample of cancer survivors recruited from 3 SEER registries and the breadth of the HINTS survey items, which allowed for a comprehensive analysis of variables that may be associated with knowledge of clinical trials. Despite these strengths, some limitations must be noted. First, although we assessed knowledge of clinical trials, we do not know whether cancer survivors who took the HINTS were knowledgeable about *specific* clinical trials (ie, those that were actively recruiting participants) versus general knowledge that clinical trials are possible options for cancer survivors. People who were knowledgeable about a specific clinical trial may have had more knowledge about clinical trials overall because they received information about what to consider when joining a clinical trial (eg, time demand, risks, benefits). Second, the question regarding clinical trial knowledge was broad and not limited to cancer-specific clinical trials. Third, it is unclear what knowing “a lot” about clinical trials meant to participants, and how that knowledge may have translated into actual enrollment in cancer clinical trials or other clinical trials beyond cancer (eg, mental health, diabetes, hypertension). Fourth, each of the 3 SEER registries had a different process for consenting cancer survivors to the HINTS-SEER survey, which may have affected those who were aware of the HINTS-SEER survey opportunity and ultimately participated. Lastly, this study represents survivors from only 3 SEER registries (Iowa, Greater Bay Area, and New Mexico), which may not be representative of all US cancer survivors.

### Future Directions

Future work should explore specific domains of clinical trial knowledge among cancer survivors (eg, where to find them and different phases of clinical trials) and areas of greatest concern regarding clinical trial participation (eg, side effects and time commitment). Additionally, more research is needed regarding patient-provider communication regarding clinical trials and how the topic is typically introduced to cancer survivors. Finally, determining which attributes of health-related videos are most likely to appeal to cancer survivors warrants further research (eg, length, content, and types of people featured in videos).

### Conclusions

In summary, sociodemographic factors and health information-seeking behaviors including engagement with online health videos were associated with clinical trial knowledge among US cancer survivors. These results suggest that enhancing digital health literacy, supporting evidence-based video education, and ensuring equitable clinician-patient discussions are promising strategies for increasing clinical trial awareness. Policies that fund digital outreach and promote equitable trial communication, along with research to identify the most effective educational approaches, will be critical to translating these findings into practice.

## Abbreviations

HINTS: Health Information National Trends Survey
IRB: Institutional Review Board
OR: odds ratio
SEER: Surveillance, Epidemiology, and End Results

## References

[1] Commaroto S, Camacho-Rivera M, Guo Y (2024). Racial and ethnic disparities in knowledge, attitudes, and invitation to participate in clinical trials among cancer survivors in the United States: an analysis of the 2020 U.S. HINTS. Prev Med Rep.

[2] (2024). About cancer clinical research. National Cancer Institute.

[3] Aiyegbusi OL, Cruz Rivera S, Kamudoni P (2024). Recommendations to promote equity, diversity and inclusion in decentralized clinical trials. Nat Med.

[4] Herremans KM, Riner AN, Winn RA, Trevino JG (2021). Diversity and inclusion in pancreatic cancer clinical trials. Gastroenterology.

[5] Javier-DesLoges J, Nelson TJ, Murphy JD (2022). An evaluation of trends in the representation of patients by age, sex, and diverse race/ethnic groups in bladder and kidney cancer clinical trials. Urol Oncol.

[6] Birhiray MN, Birhiray RE (2023). Practical strategies for creating diversity, equity, inclusion, and access in cancer clinical research: DRIVE. Blood Adv.

[7] Yadav S, Todd A, Patel K (2022). Public knowledge and information sources for clinical trials among adults in the USA: evidence from a Health Information National Trends Survey in 2020. Clin Med (Lond).

[8] Rivera-Díaz M, García-Romero AN, Ayala-Marín AM (2020). Knowledge, motivations and concerns about participation in breast cancer clinical trials in Puerto Rico. J Health Dispar Res Pract.

[9] Langford A, Resnicow K, An L (2010). Clinical trial awareness among racial/ethnic minorities in HINTS 2007: sociodemographic, attitudinal, and knowledge correlates. J Health Commun.

[10] Santana S, Brach C, Harris L (2021). Updating health literacy for healthy people 2030: defining its importance for a new decade in public health. J Public Health Manag Pract.

[11] Langford AT, Ellis KR, Orellana K, France BM, Buderer N (2023). Self-efficacy to get cancer-related information or advice. J Cancer Educ.

[12] Davis SN, O’Malley DM, Bator A, Ohman-Strickland P, Hudson SV (2021). Correlates of information seeking behaviors and experiences among adult cancer survivors in the USA. J Cancer Educ.

[13] Curbow B, Fogarty LA, McDonnell K, Chill J, Scott LB (2004). Can a brief video intervention improve breast cancer clinical trial knowledge and beliefs?. Soc Sci Med.

[14] Massett HA, Dilts DM, Bailey R (2017). Raising public awareness of clinical trials: development of messages for a national health communication campaign. J Health Commun.

[15] Harris PA, Scott KW, Lebo L, Hassan N, Lightner C, Pulley J (2012). ResearchMatch: a national registry to recruit volunteers for clinical research. Acad Med.

[16] About clinicaltrials.gov. ClinicalTrials.gov.

[17] Christy SM, Livingstone AS, Byrne MM (2022). Feasibility, acceptability, and effectiveness of a decision aid versus an informational website to promote clinical trial decision-making among cancer patients: a pilot randomized controlled trial. Patient Educ Couns.

[18] Pathak S, George N, Monti D, Robinson K, Politi MC (2019). Evaluating adaptation of a cancer clinical trial decision aid for rural cancer patients: a mixed-methods approach. J Cancer Educ.

[19] Morgan SE, Peng W, Occa A (2022). Tailored messages about research participation: using an interactive information aid to improve study recruitment. J Cancer Educ.

[20] Langford AT, Larkin K, Resnicow K, Zikmund-Fisher BJ, Fagerlin A (2017). Understanding the role of message frames on African-American willingness to participate in a hypothetical diabetes prevention study. J Health Commun.

[21] Dreyer NA, Blackburn SC, Mt-Isa S (2015). Direct-to-patient research: piloting a new approach to understanding drug safety during pregnancy. JMIR Public Health Surveill.

[22] Plante TB, Gleason KT, Miller HN (2020). Recruitment of trial participants through electronic medical record patient portal messaging: a pilot study. Clin Trials.

[23] Sherman SE, Langford AT, Chodosh J, Hampp C, Trachtman H (2022). Use of patient portals to support recruitment into clinical trials and health research studies: results from studies using MyChart at one academic institution. JAMIA Open.

[24] Ellis PM, Butow PN, Tattersall MHN (2002). Informing breast cancer patients about clinical trials: a randomized clinical trial of an educational booklet. Ann Oncol.

[25] Yarbrough K (2010). Patient and family fact sheet. Clinical trial research: an overview for patients and families. Neurologist.

[26] Tan NH, Lafeber M, Sablerolles RSG (2024). Evaluation of a group-based online informed consent conversation (eConsent) in participants from a low-risk vaccination clinical trial. Trials.

[27] Mayhew M, Leo MC, Vollmer WM, DeBar LL, Kiernan M (2020). Interactive group-based orientation sessions: a method to improve adherence and retention in pragmatic clinical trials. Contemp Clin Trials Commun.

[28] Kumar G, Kim J, Farazi PA, Wang H, Su D (2022). Disparities in awareness of and willingness to participate in cancer clinical trials between African American and White cancer survivors. BMC Cancer.

[29] Cho Y, Shang S, Zhou W (2023). Comorbidities were associated with cancer clinical trial discussion and participation: findings from the Health Information National Trends Survey-Surveillance, Epidemiology, and End Results Program (2021). J Clin Epidemiol.

[30] Nelson DE, Kreps GL, Hesse BW (2004). The Health Information National Trends Survey (HINTS): development, design, and dissemination. J Health Commun.

[31] About HINTS. National Cancer Institute.

[32] Survey instruments. National Cancer Institute.

[33] (2022). Health Information National Trends Survey 5 (HINTS 5): HINTS-SEER methodology report. https://hints.cancer.gov/docs/methodologyreports/HINTS_SEER_MethodologyReport.pdf.

[34] (2024). Social media fact sheet. Pew Research Center.

[35] Garg A, Nyitray AG, Roberts JR (2024). Consumption of health-related videos and human papillomavirus awareness: cross-sectional analyses of a US National Survey and YouTube from the urban-rural context. J Med Internet Res.

[36] Zhou F, Xu X, Trajcevski G, Zhang K (2021). A survey of information cascade analysis: models, predictions, and recent advances. ACM Comput Surv.

[37] Nutbeam D, Lloyd JE (2021). Understanding and responding to health literacy as a social determinant of health. Annu Rev Public Health.

[38] Nutbeam D (2000). Health literacy as a public health goal: a challenge for contemporary health education and communication strategies into the 21st century. Health Promot Int.

[39] Urstad KH, Andersen MH, Larsen MH, Borge CR, Helseth S, Wahl AK (2022). Definitions and measurement of health literacy in health and medicine research: a systematic review. BMJ Open.

[40] Byrne MM, Tannenbaum SL, Glück S, Hurley J, Antoni M (2014). Participation in cancer clinical trials: why are patients not participating?. Med Decis Making.

[41] Langford AT, Resnicow K, Dimond EP (2014). Racial/ethnic differences in clinical trial enrollment, refusal rates, ineligibility, and reasons for decline among patients at sites in the National Cancer Institute’s Community Cancer Centers Program. Cancer.

[42] Hoadley A, Fleisher L, Kenny C (2024). Exploring racial disparities in awareness and perceptions of oncology clinical trials: cross-sectional analysis of baseline data from the mychoice study. JMIR Cancer.

[43] Langford AT (2020). Health communication and decision making about vaccine clinical trials during a pandemic. J Health Commun.

[44] Jiang S, Hong YA (2021). Clinical trial participation in America: the roles of eHealth engagement and patient-provider communication. Digit Health.

[45] Tan SSL, Goonawardene N (2017). Internet health information seeking and the patient-physician relationship: a systematic review. J Med Internet Res.

[46] J JD, D WA, A CK, J S (1995). A comprehensive model of information seeking: tests focusing on a technical organization. Sci Commun.

[47] Jia X, Pang Y, Liu LS (2021). Online health information seeking behavior: a systematic review. Healthcare (Basel).

[48] Wissler Gerdes EO, Nash SH, Vanderpool RC, Van Blarigan EL, Meisner ALW, Senft Everson N (2025). Clinical trial knowledge, discussion, and participation among cancer survivors: a HINTS-SEER study. Patient Educ Couns.

[49] Langford A, Loeb S (2019). Perceived patient-provider communication quality and sociodemographic factors associated with watching health-related videos on YouTube: a cross-sectional analysis. J Med Internet Res.

